# The effect of a chrysanthemum water extract in protecting the retina of mice from light damage

**DOI:** 10.1186/s12906-022-03701-2

**Published:** 2022-08-26

**Authors:** Yibo Gong, Xuechun Wang, Yuchuan Wang, Peng Hao, Hao Wang, Yatu Guo, Wei Zhang

**Affiliations:** 1 Tianjin Eye Hospital, Tianjin Eye Institute,Tianjin Key Laboratory of Ophthalmology and Visual Science, 4 Gansu Road, Tianjin, China; 2 Tianjin Medical University, 22 Qixiangtai Road, Tianjin, China; 3 Nankai University Affiliated Eye Hospital, 4 Gansu Road, Tianjin, China; 4 State Key Laboratory of Food Nutrition and Safety, Tianjin University of Science and Technology, Tianjin, China

**Keywords:** Light-damaged retina, Chrysanthemum, Antioxidant, ROS, NF-кB, TNF-α, Apoptosis

## Abstract

**Background:**

Oxidative stress can induce age-related diseases. Age-related retinal diseases, such as age-related macular degeneration (AMD), are difficult to cure owing to their complicated mechanisms. Although anti-neovascular therapeutics are used to treat wet AMD, vision cannot always be completely restored, and disease progression cannot always be inhibited. Therefore, determining a method to prevent or slow retinal damage is important. This study aimed to investigate the protective effect of a chrysanthemum water extract rich in flavone on the oxidatively stressed retina of mice.

**Methods:**

Light damage was induced to establish oxidative stress mouse models. For in vitro experiments, ARPE-19 cells were cultured and divided into four groups: control, light-damaged, and low- and high-dose chrysanthemum extract. No treatment was administered in the control group. The light-damaged and low- and high-dose chrysanthemum extract groups were exposed to a similar white light level. The chrysanthemum extract was added at a low dose of 0.4 mg/mL or a high dose of 1.0 mg/mL before cell exposure to 2500-lx white light. Reactive oxygen species (ROS) level and cellular viability were measured using MTT and immunofluorescence staining. For in vivo experiments, C57BL/6 J mice were divided into the same four groups. Low- (0.23 g/kg/day) and high-dose (0.38 g/kg/day) chrysanthemum extracts were continuously intragastrically administered for 8 weeks before mouse exposure to 10,000-lx white light. Retinal function was evaluated using electroretinography. In vivo optical coherence tomography and in vitro haematoxylin and eosin staining were performed to observe the pathological retinal changes in each group after light damage. Fluorescein fundus angiography of the arteriovenous vessel was performed, and the findings were analysed using the AngioTool software. TUNEL immunofluorescence staining was used to assess isolated retinal apoptosis.

**Results:**

In vitro, increased ROS production and decreased ARPE-19 cell viability were found in the light-damaged group. Improved ARPE-19 cell viability and reduced ROS levels were observed in the chrysanthemum extract treatment groups. In vivo, dysfunctional retinas and abnormal retinal structures were found in the light-damaged group, as well as increased apoptosis in the retinal ganglion cells (RGCs) and inner and outer nuclear layers. The apoptosis rate in the same layers was lower in the chrysanthemum extract treatment groups than in the light-damaged group. The production of antioxidant enzymes, including superoxide dismutase (SOD), catalase (CAT), and glutathione peroxidase (GSH-Px), increased in the treatment groups. NF-κB in the nucleus and TNF-α were more highly expressed in the light-damaged group than in the low- and high-dose chrysanthemum extract groups.

**Conclusions:**

Light damage-induced retinal oxidative stress can lead to ROS accumulation in the retinal tissues. Herein, RGC and photoreceptor layer apoptosis was triggered, and NF-κB in the nucleus and TNF-α were highly expressed in the light-damaged group. Preventive chrysanthemum extract administration decreased ROS production by increasing SOD, CAT, and GSH-Px activities and reversing the negative changes, demonstrating a potential protective effect on the retina.

## Background

Oxidative stress has a strong correlation with age-related diseases; correspondingly, antioxidant foods, materials, and medicines have been a common treatment for preventing some diseases associated with ageing. Age-related macular degeneration (AMD) is a common disease in developed countries that tends to occur in young individuals. According to a previous meta-analysis, the number of patients with AMD could reach 0.3 billion by 2040 [1]. AMD is characterised by the aggregation of drusen and destruction of the retinal pigment epithelium (RPE) layer in the early period, which gradually develops into the dry type with geographic atrophy or the wet type with neovascularisation [2, 3]. The mechanism of AMD is not completely clear, and some studies have shown that exposure to high-intensity light or a lack of antioxidant food in the daily diet could lead to excessive reactive oxygen species (ROS) production [4–6]. ROS that cannot be metabolised leads to simultaneously increased apoptosis and NF-_K_B and TNF-α upregulation [7]. Thus, administration of antioxidant enzymes, such as superoxide dismutase (SOD), catalase (CAT), and glutathione peroxidase (GSH-Px), which inhibit the production of ROS, could be a preventive therapy for age-related diseases.

Chrysanthemum, which has been considered a common herb in traditional Chinese medicine for thousands of years and can be consumed directly with warm water in daily life, contains antioxidant materials, especially flavonoid micromolecules, that are useful in scavenging oxygen free radicals caused by light damage [8–10]. Experiments to confirm and describe the effect of chrysanthemum extracts on the antioxidant activity in the retina and the related mechanisms are rare. Thus, our primary aim was to investigate the protective effect of a chrysanthemum water extract against damage induced by oxidative stress on the retina and to further identify the possible mechanistic pathways.

## Methods

### Chrysanthemum water extraction

Chrysanthemum produced in Hangzhou, China was used in this study, and the petals were the main parts used for water extraction. Chrysanthemum petals were soaked in water at a weight eight times that of the petals and then boiled for 0.5 h. The liquid was stored in bottles, and the residue was boiled again. This process was repeated three times, and all the liquids obtained were combined. The combined liquid was concentrated to low and high concentrations of extract (1 g of chrysanthemum extract was obtained from 4.255 g of material) [11]. The total content of flavonoids in the chrysanthemum reached an effective concentration of 5.0%, according to technical specifications for inspection and evaluation of healthy food.

### Cell experiments

#### Cell culture

ARPE-19 cells (Beijing Dingguo Biotechnology, Beijing, China) were cultured in DMEM/F12 (HyClone, USA) containing 10% foetal bovine serum, 100 U/mL penicillin, and 100 mg/mL streptomycin and incubated in 5% CO2 at a constant temperature of 37 °C. The cells were grown to the exponential phase before use in the experiments.

#### Toxicity of the chrysanthemum extract to the ARPE-19 cells

The concentration of the chrysanthemum extract that was safe and non-toxic to the ARPE-19 cells was assessed using the MTT assay. The ARPE-19 cells (approximately 10 × 103 cells/well) were plated in 96-well plates at 100 μL/well. After the cells were grown to the exponential phase, the chrysanthemum extracts at concentrations of 0.2, 0.4, 0.8, 1.0, 1.2, 1.5, 2.00, and 5.00 mg/mL were added to the 96-well plates and incubated for 24 h. At the end of the culture period, 20 μL MTT (5 mg/mL) solution was added to the cells, followed by incubation in a CO2 incubator for 4 h. The MTT solution was discarded, and 150 μL of DMSO solution was added. After the cells were fully dissolved, the cell activity was evaluated by measuring the absorbance (A value) at a wavelength of 492 nm. Based on the results of this assay, a concentration deemed safe was determined and used in the subsequent experiments.

#### Light-induced damage to the RPE cells

The cells were digested with trypsin to prepare a suspension and then plated on a culture plate. They were irradiated directly with LED white light with an intensity of 2500 ± 500 lx for 24 h in the light-damaged group (LD group), low-dose chrysanthemum extract group (LC group), and high-dose chrysanthemum extract group (HC group) during the exponential growth phase [12].

#### Measurement of ROS levels in the four groups

Intracellular ROS levels were measured using DCFH-DA probes (Gibco, USA). A suspension of ARPE-19 cells (1.5 × 10^5^/well) was seeded into a 6-well plate and cultured for 24 h. The cell culture medium was replaced with DCFH-DA, and the cells were cultured at 37 °C for 20 min. The ImageJ software (National Institutes of Health, https://imagej.en.softonic.com/) was used to analyse the fluorescence intensity.

### Animals

#### Group division and chrysanthemum extract treatment

Male C57BL/6 J mice aged 8 weeks (*n* = 32) were obtained from SPF Biotechnology Co., Ltd. (Beijing, China). The mice were raised in the following conditions: 12/12 h light/dark cycles, temperature of 23 ± 2 °C, and relative humidity of 55%, at the Tianjin Eye Institute; food and water were provided freely. They were randomly divided into four groups: control (*n* = 8), LD (*n =* 8), LC (*n =* 8), and HC groups (*n =* 8). After the chrysanthemum extract was concentrated and dried, the obtained solid was crushed into a powder. The powder was suspended in 0.9% saline for intragastric administration, which was conducted via insertion of an intragastric tube with a diameter of 1.25 mm and a length of no more than 3 cm into the digestive tract. The effective concentration ranged from 0.23 (low dose) to 0.38 g/kg/day (high dose). Intragastric administration (0.2 mL/day) was performed continuously for 8 weeks. The dose and concentration induced no obvious uncomfortable response in our experiments, which was based on the study by Lumeng et al. on the protective effect of chrysanthemum in mice with Parkinson’s disease [13].

#### Light damage induction

After 8 weeks of intragastric administration, the light injury mode was established as follows: the mice in the LD, LC, and HC groups were treated with continuous white light at 10000 lx for 7 days under the condition of dilating pupils using 0.5% tropicamide phenylephrine eye drops (Mydrin, Santen, Japan) 4 h/day for 28 h in total. Each mouse was separated by a transparent box during light exposure to avoid injury caused by crowding.

#### Electroretinogram (ERG)

After 12 h of dark adaptation, the mice were anaesthetised via inhalation of 2% isoflurane and placed on an experimental table at temperature of 40 °C. Both eyes were dilated, and an eye gel was used to keep the cornea transparent. Three types of electrode were used. The reference electrode was connected to the head, annular corneal electrode to the eyeball, and ground electrode to the tail of the mouse [14]. ERG was used to record the a-, b-, and Ops waves after a flash stimulus of 0.01 cds/m^2^ and 3.0 cds/m^2^ in scotopic adaptation. After 10 minutes of photopic adaptation, a- and b-waves were evoked by a flash of 3.0 cds/m^2^ in the photopic mode (RETI-Port 21, Roland, Germany).

#### Optical coherence tomography (OCT)

The mice were placed on the animal experimental platform after being anaesthetised via inhalation of 2% isoflurane; 0.5% tropicamide phenylephrine eye drops (Mydrin, Santen) were used to dilate the pupils, and a carbomer eye gel was used to keep the corneas transparent. Phoenix eye testing equipment for animals (model: Micron IV, Phoenix Research Labs) was used to scan the retina in vivo and obtain morphological images of each layer. Furthermore, the retinal thickness of the inner nuclear layer (INL) and outer nuclear layer (ONL) of the four groups was measured using a software and compared manually from an area of − 600 to an area of 600 μm from the optic nerve.

#### Fluorescein fundus angiography (FFA)

Approximately 2 mL of 2% sodium fluorescein was intraperitoneally injected at the end of OCT [15]. The arterial, venous, and arteriovenous phases were clearly observed under the same gain value. The AngioTool (version 0.6a; https://ccrod cancer.gov/confluence/display/ROB2/Downloads), a free-to-download software, was used to analyse the parameters consisting of the vessel area, vessel area percentage, and total number of junctions. This application can be easily used, and the detailed method has been described by Zudaire et al. [16].

ERG, OCT, and FFA were performed step by step when the pupil was dilated enough. ERG was first conducted as soon as possible, and repeat examination was avoided because this would make the outcome incorrect. The ERG room was dark and absolutely quiet. When ERG was finished, the same mouse was transferred to the OCT and FFA rooms. The two examinations were completed using a Phoenix eye testing equipment for animals (model: Micron IV, Phoenix Research Labs) [17]. All abovementioned progress was measured in vivo; the mice were kept alive; the cornea was specifically evaluated; a carbomer eye gel was continuously added to the surface of the eye to prevent opacities from occurring; and no fewer than three experienced operators were needed.

#### Haematoxylin and eosin (HE) and TUNEL staining

To obtain blood only after 3 days when sodium fluorescein was excreted completely, we sacrificed all the mice via inhalation of isoflurane, collected the ophthalmic blood vessels, and removed the eyeballs simultaneously. Retinal tissue could be a better option for measuring SOD, CAT, and GSH-Px activities; however, this will require too many mice to obtain enough materials, so blood from the optic artery was considered an economical sample. After 12 h of fixation of the eyeballs in formalin, part of the cornea was cut off and fixed in fresh formalin for another 8 h. After dehydration and paraffin liquid immersion at 40 °C for 6 h, the eyeballs were completely embedded in paraffin blocks. The paraffin blocks were cut into 4-μm-thick parasagittal sections. The sections were then subjected to HE staining, TUNEL immunofluorescence staining (TUNEL fluorescence kit provided by Dalian Meilun Biotechnology Co., Ltd., Dalian, China), and other immunofluorescence techniques [18, 19]. In each group, four eyeballs from different mice were analysed to count the apoptotic cells. At least four sections were counted from each eye; thus, 16 sections were used in each group and subjected to TUNEL immunostaining. To calculate the apoptosis rate, the tunel (+) cells in the retina were counted manually with Image J software (National Institutes of Health, https://imagej.en.softonic.com/).

#### Immunofluorescence staining of the retina

The paraffin slices (4 μm) were performed with antigen repair and blocked with 5% BSA for 1 h. Then the slices were incubated with the antibodies of NF-κB (1:200, SAB4300554, Sigma-Aldrich LLC, Germany) and TNF-α (1:200, 17,590-1-AP, Proteintech Group, Inc., Wuhan, China) at 4 °C overnight. The secondary antibodies coupled with Alexa Fluor 488 (1: 1000, SA00013-2, Proteintech Group, Inc.) were used to incubated with slices for 2 h. The nucleus was stained with DAPI for 5 min. Finally, the slices were imaged by Leica SP8 confocal microscope with 20× objective. The fluorescence intensity was detected using the ImageJ software.

#### Antioxidant enzyme assays

All the groups of blood collected from the ophthalmic artery were centrifuged at 4 °C, 3000 r/min for 10 min to obtain the serum. They were kept at − 80 °C to measure the enzymatic activities of SOD, CAT, and GSH-Px. The method of examination followed the instructions of the SOD, CAT, and GSH-Px assay kits (WST-1, Nanjing Jiancheng Bioengineering Institute).

### Statistical methods

All parameters, including ROS expression, cell viability, a- and b-wave amplitude on ERG, retinal thickness, vessel area, vessel percentage, total number of junctions, apoptosis rate, antioxidant enzyme (SOD, CAT, and GSH-Px) content in the blood, and NF-κB and TNF-α expression density, were presented as *x ± s.* One-way ANOVA and the SPSS software (version 22.0, IBM Corp., Chicago, USA) were used to evaluate all parameters. Differences with a *P*-value of < 0.05 were considered significant. *P*-values of < 0.01 indicated a more obvious difference.

## Results

### Cell examinations

#### Toxicity of chrysanthemum to the ARPE-19 cells

The MTT assay showed that the cell viability rate in the control group was 99.98%. The cell viability rates were 97.64, 98.52, 96.18, 96.24, 94.42, and 90.65% after treatment with 0.2, 0.4, 0.8, 1.0, 1.2, and 1.5 mg/mL of chrysanthemum extract, respectively. At a concentration of 2.00 mg/mL, the chrysanthemum extract significantly inhibited the viability of the ARPE-19 cells, and the cell viability rate decreased to 88.15% (*P* < 0.05). At a concentration of 5 mg/mL, the cell viability rate decreased to 64.66% (*P* < 0.001). Therefore, a chrysanthemum extract concentration of no more than 1.2 mg/mL was selected for the subsequent experiments. The LC group was treated with 0.4 mg/mL of chrysanthemum extract, while the HC group was treated with 1.0 mg/mL of chrysanthemum extract.

#### Effect of the chrysanthemum extract on the viability and ROS production in the ARPE-19 cells after light damage

**Fig. 1:**
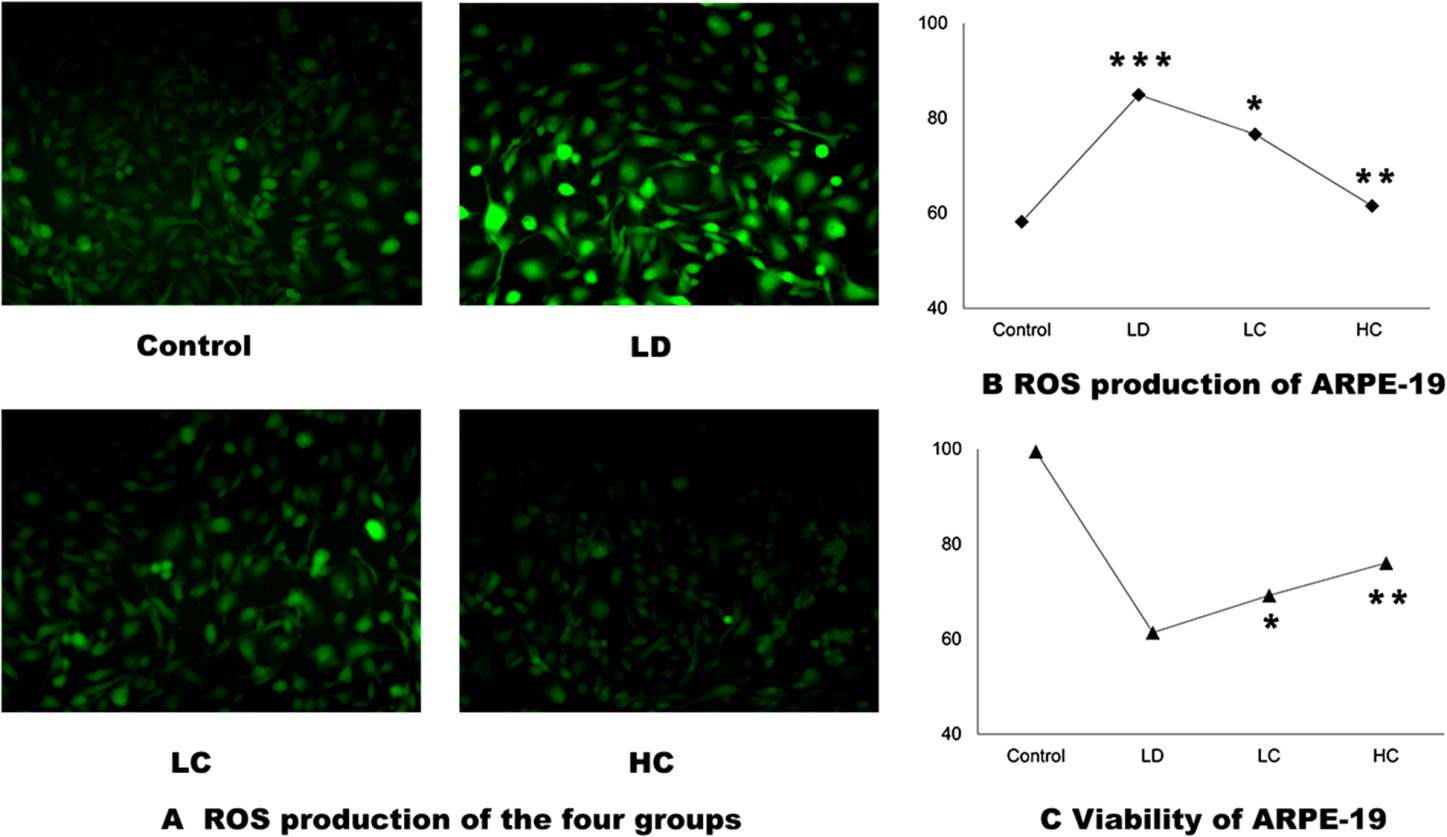
ROS production (**a**, **b**) and viability (**c**) of the ARPE-19 cells. **a**, **b**: The ImageJ software was used to measure the intensity of the fluorescence. The rate of production of ROS in the LD group (84.94 ± 3.29%) increased by 45.92% (*P < 0.001*) compared with that in the control group (58.21 ± 6.46%); the rate in the LC (76.62 ± 4.40%) and HC (61.56 ± 2.02%) groups decreased by 10.5% (*P < 0.05*) and 27.53% (*P < 0.0001*), respectively, compared with that in the LD group. **c**: After treatment with the chrysanthemum extract before light damage, the cell viability rate in the LC and HC groups increased to 69.22 and 76.04%, respectively, compared with that in the LD group (*P <* 0.05 and *P <* 0.001); the data were analysed using one way-ANOVA. *P < 0.05, **P < 0.01, ***P < 0.0001

The cell viability rate in the LD group decreased to 61.43% (*P <* 0.001) after light exposure at 2500 lx for 24 h. After treatment with the chrysanthemum extract, the cell viability rate in the LC and HC groups increased to 69.22 and 76.04%, respectively, compared with that in the LD group (*P <* 0.05 and *P <* 0.001). The rate of production of ROS in the LD group (84.94 ± 3.29%) increased by 45.92% (*P <* 0.001) compared with that in the control group (58.21 ± 6.46%); the rate in the LC (76.62 ± 4.40%) and HC (61.56 ± 2.02%) groups decreased by 10.5% (*P <* 0.05) and 27.53% (*P <* 0.0001), respectively, compared with that in the LD group (Fig. 1a–c).

### Animal examinations

#### OCT and HE staining

**Fig. 2:**
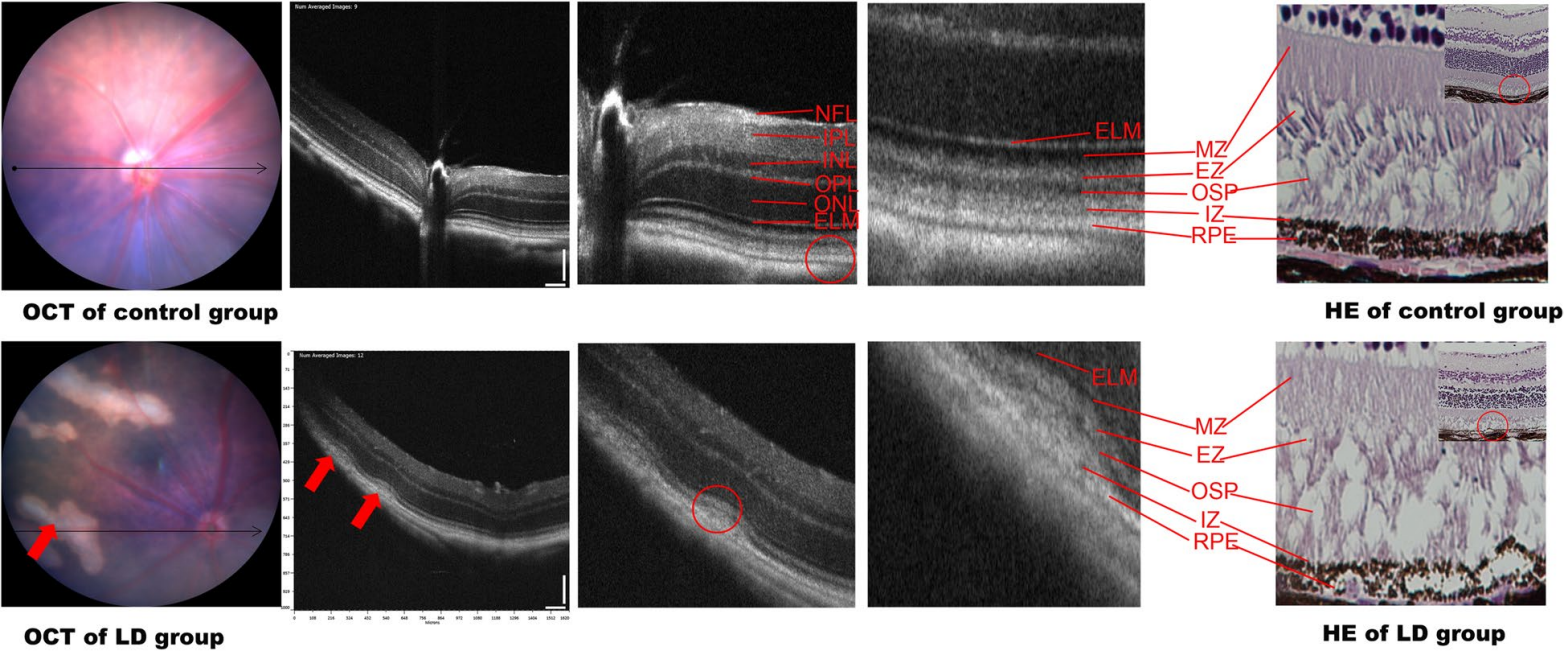
Retinal structure of the control (**a**) and LD (**b**) groups on OCT. **a**: NFL: nerve fibre layer. IPL: inner plexiform layer. INL: inner nuclear layer. OPL: outer plexiform layer. ONL: outer nuclear layer; ELM: external limiting membrane; MZ: myoid zone. EZ: ellipsoid zone. OSP: outer segment of the photoreceptors. IZ: interdigitation zone. RPE: retinal pigment epithelium. **b**: The red arrow shows the signal of high reflection between the RPE and IZ layers, corresponding to the destroyed PRE, IZ, OSP, and EZ layers shown on HE staining. The results of OCT and HE staining were similar to those of AMD in the early phase

**Fig. 3:**
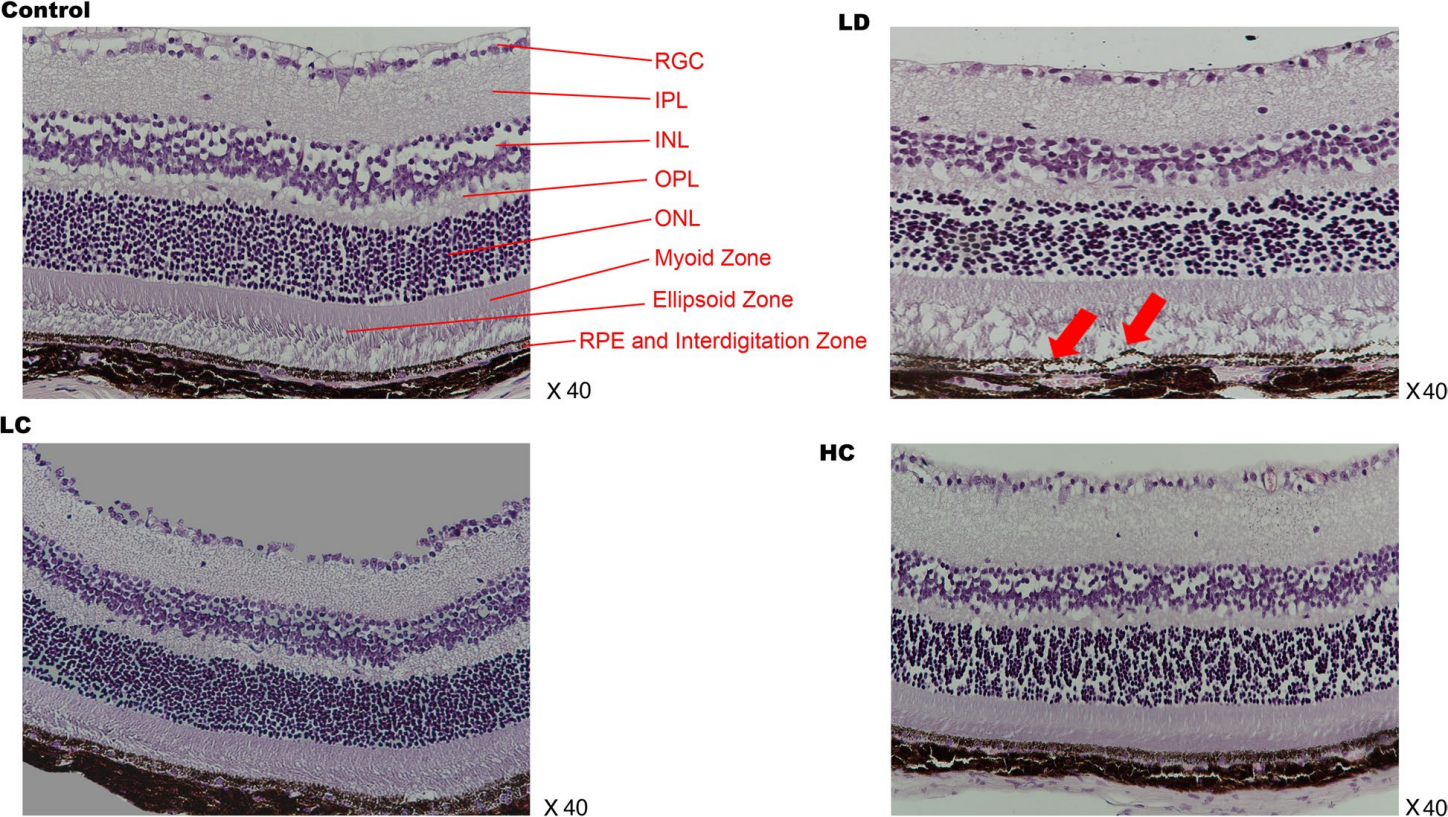
HE-stained images of the four groups. HE (×40): The destroyed RPE and photoreceptors of the retina in the LD group are marked with red arrows. No significant deformation is observed in the LC and HC groups after light damage

**Fig. 4:**
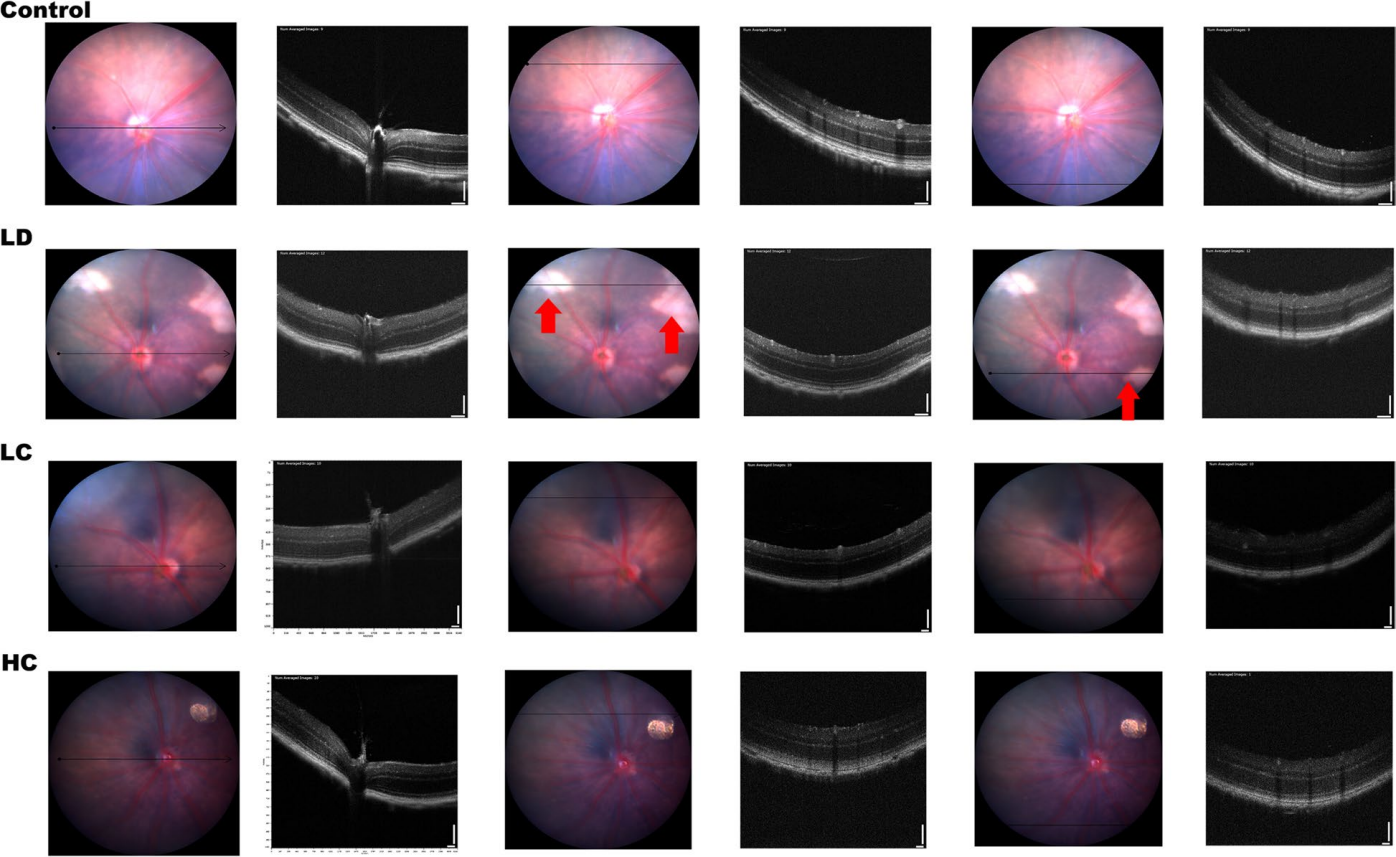
OCT and FFA images in each group. OCT: Compared with those in the LD group, the retinal structures in the LC and HC groups maintained a relative integrity and had no signals of high reflection in the RPE layer. The red arrow shows the pathological position caused by light damage

**Fig. 5:**
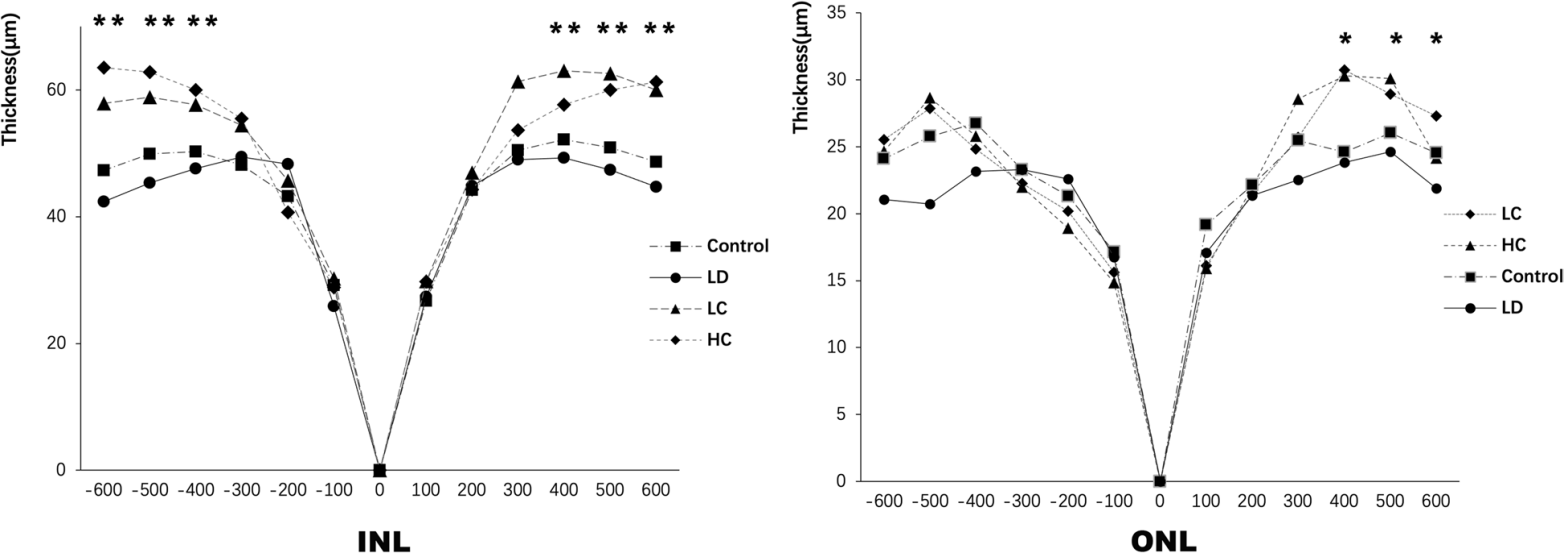
Thickness of the INL and ONL. The peri-retinal zone of the INL was significantly thinner when analysed from 300 to 600 μm from the optic nerve. This tendency became more obvious from − 300 to − 600 μm and from 300 to 600 μm from the optic nerve. The detailed data are presented in Table 1

OCT showed that a signal of high reflection of an arch shape was observed between the RPE and interdigitation zone layer in the LD group, which also led to deformations in the outer segment of the photoreceptors (OSP), ellipsoid zone (EZ) layer, and myoid zone (MZ) layer. In addition, the damaged structure of the retina observed on HE staining was similar to that observed on OCT (Fig. 2). The retinal structure depicted on OCT and HE staining significantly improved in the LC and HC groups compared with that in the LD group (Figs. 3 and 4). The ONL from − 600 to 600 μm from the optic nerve in the LD group was much thinner than that in the control group; however, after treatment with the chrysanthemum extract before light damage, the layer in the LC and HC groups was much thicker than that in the LD group, especially from − 600 to − 300 μm and from 300 to 600 μm from the optic nerve (*P* < 0.05). The decreased thickness of the INL also illustrated that the peri-retinal area was markedly thicker in the LC and HC groups than in the LD group (*P* < 0.05) (Fig. 5 and Table 1).

**Table 1:** Thickness of the INL and ONL in the four groups

ONL													
***μm***	−600	−500	− 400	−300	− 200	− 100	0	100	200	300	400	500	600
Control	47.33 ±3.05	49.92 ±2.65	50.30 ±2.65	48.15 ±1.88	43.20 ±0.58	29.19 ±4.52	0	26.72 ±4.42	44.19 ±3.89	50.50 ±1.75	52.21 ±2.96	50.88 ±3.80	48.63 ±1.99
LD	42.41 ±2.20	45.34 ±1.31	47.60 ±2.45	49.44 ±1.85	48.27 ±3.87	25.88 ±1.64	0	27.37 ±2.03	44.86 ±3.79	49.04 ±3.02	49.30 ±3.90	47.38 ±2.41	44.70 ±2.04
LC	57.88 ±2.70 **	58.86 ±3.10 **	57.68 ±3.58 **	54.40 ±3.27 **	45.73 ±3.42	30.24 ±1.43	0	29.86 ±3.45	46.96 ±5.74	61.35 ±7.21 **	63.06 ±4.95 **	62.64 ±5.52 **	60.01 ±5.57 **
HC	62.49 ±3.06 **	62.85 ±5.02 **	59.99 ±4.59 **	55.52 ±3.26 **	40.71 ±4.09	28.85 ±3.64	0	29.77 ±2.29	44.27 ±1.57	53.66 ±3.88 **	57.67 ±1.96 **	59.99 ±1.72 **	61.25 ±4.40 **
INL													
***μm***	−600	−500	−400	−300	−200	−100	0	100	200	300	400	500	600
Control	24.11 ±3.23	25.78 ±3.18	26.77 ±3.45	23.28 ±3.68	21.32 ±3.55	17.15 ±4.22	0	19.17 ±3.17	22.14 ±1.98	25.47 ±2.59	24.61 ±3.34	26.06 ±1.33	24.55 ±1.31
LD	21.07 ±2.81	20.73 ±4.16	23.16 ±2.96	23.29 ±1.54	22.60 ±1.83	16.76 ±1.27	0	*17.07* ±2.27	21.35 ±2.86	22.54 ±2.52	23.84 ±1.59	24.64 ±1.72	21.90 ±2.68
LC	25.54 ±3.62	27.87 ±2.84∗	24.83 ±2.11	22.27 ±1.63	20.19 ±3.74	15.63 ±2.13	0	16.13 ±2.28	21.56 ±3.97	25.68 ±3.49	30.74 ±3.86 *	28.93 ±3.18 *	27.29 ±5.80
HC	24.66 ±4.16	28.67 ±3.71∗	25.8 ±3.12	22 ±1.70	18.91 ±2.02	14.86 ±1.40	0	15.92 ±2.22	21.96 ±3.46	28.56 ±2.63∗	30.32 ±2.03∗	30.10 ±2.32∗	24.18 ±1.21

The thickness of the layers in the four groups from −600 to 600 μm from the optic nerve was measured, and the data were analysed using ANOVA; ^*^P < 0.05, ^**^P < 0.01

#### Amplitude and implicit time on ERG

**Fig. 6:**
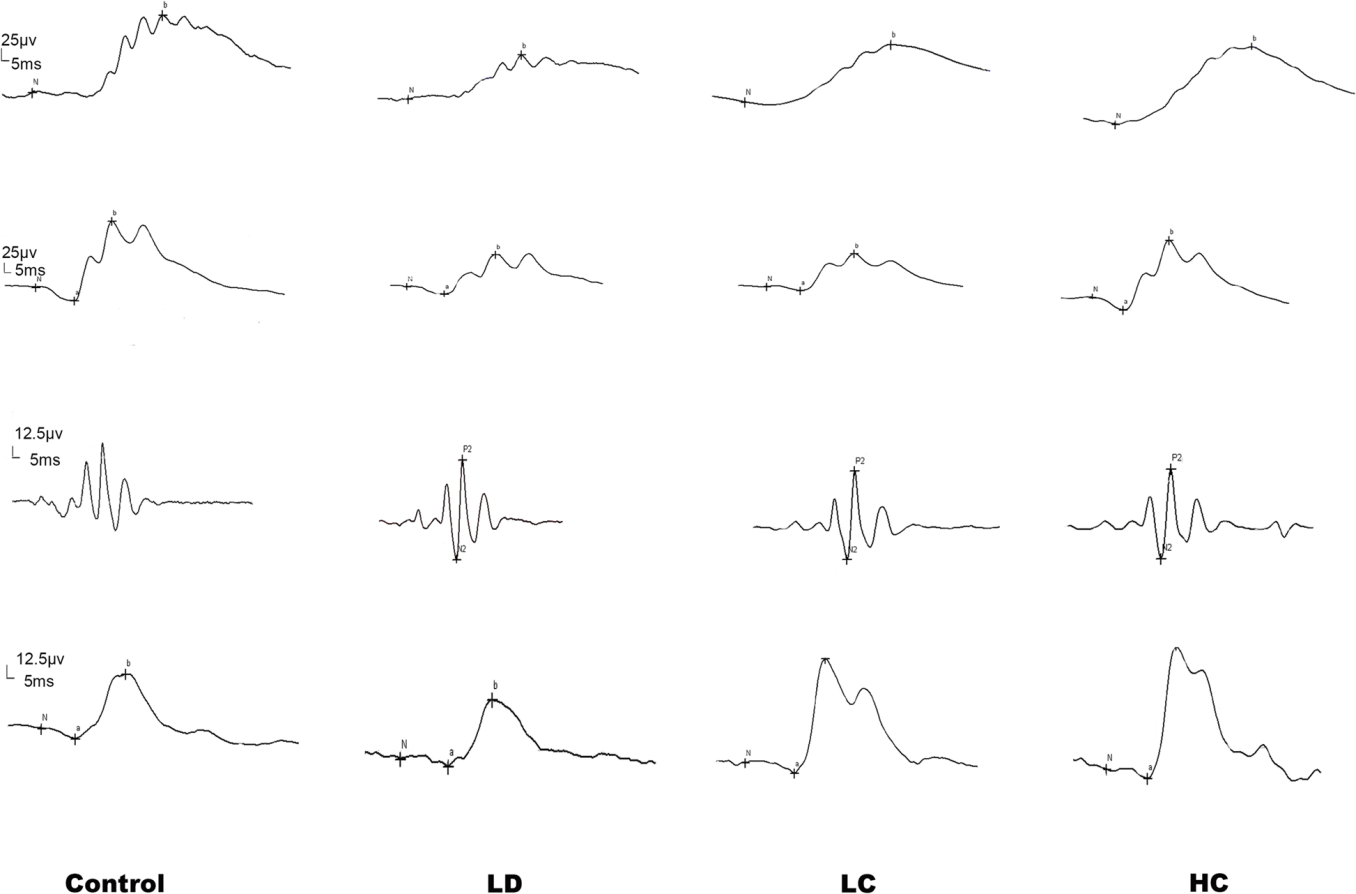
Recorded images of the full-field ERG waves in the scotopic and photopic conditions. The first trough is considered as the a-wave and the first peak as the b-wave; the amplitude of the b-wave is valued from the trough point to the peak. Ops is measured under the stimulation of 3.0 cds/m^2^ and filtered out from 20 to 40 Hz. The ruler of the amplitude and implicit time was 12.5 μv and 5 ms, respectively

**Fig. 7:**
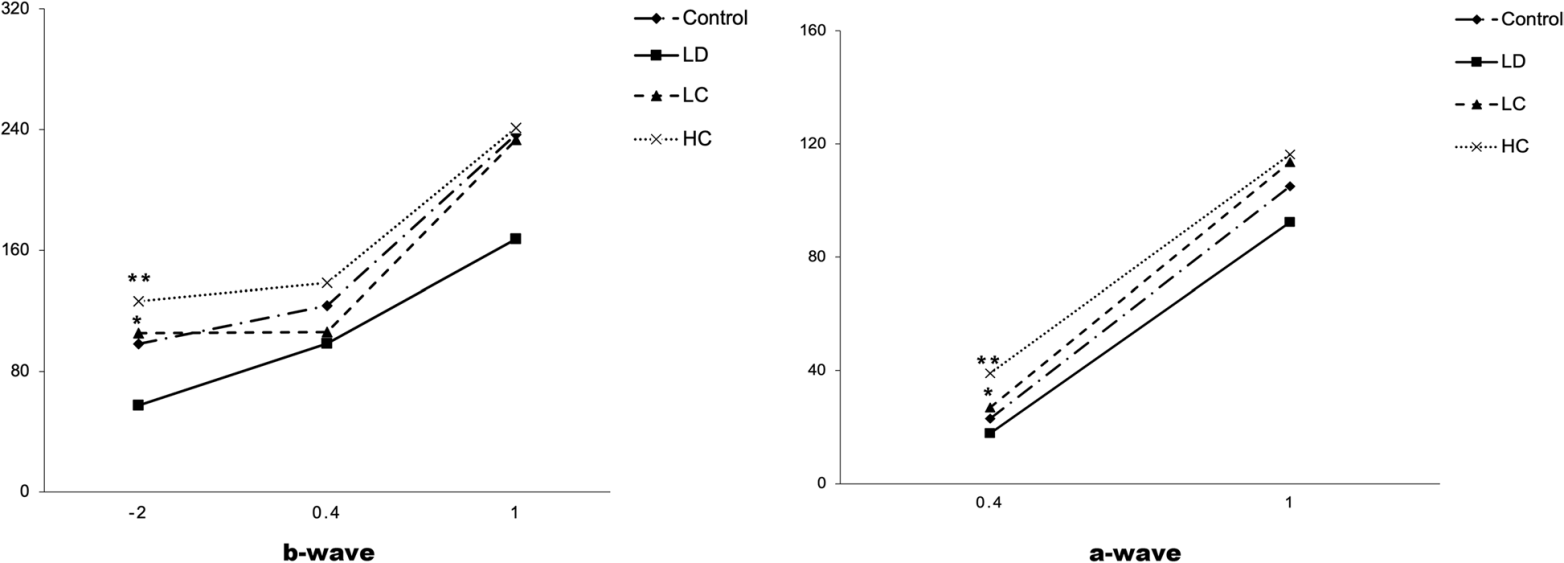
Amplitude of the a- and b-waves under the scotopic condition. **a** (scotopic): Amplitude of the b-wave under the scotopic condition of 0.01, 3.0, and 10.0 cds/m^2^. The LC group had a significantly higher amplitude than the LD group (*P* < 0.05) and the HC group (*P* < 0.01) at the stimulation of 0.01 cds/m^2^. **b**: Amplitude of the a-wave under the scotopic condition of 3.0 and 10.0 cds/m^2^. The LC group had a significantly higher amplitude than the LD group (^*^P < 0.05) and the HC group (^**^P < 0.01) at the stimulation of 3.0 cds/m^2^

The amplitude of the b-wave evoked by the stimuli of 0.01 cds/m^2^ in the scotopic response in the LD group was 41.6% lower than that in the control group; however, the amplitude in the LC and HC groups was 83.5 and 120.6% higher than that in the LD group, respectively (*P* < 0.05 for the LC group, *P* < 0.01 for the HC group). Under the 3.0 cds/m^2^ dark reaction, the a-wave amplitude evoked by the stimuli of 3.0 cds/m^2^ in the scotopic response was 22% lower in the LD group than in the control group; that in the LC group increased by 50.8%; and that in the HC group increased by 118.5%. The amplitude of the a-wave evoked by the stimuli of 3.0 cds/m^2^ in the photopic response was significantly higher in the HC group than in the LD group (*P* < 0.05) (Figs. 6 and 7). The amplitude and implicit time data are presented in Table 2.

**Table 2:** Amplitude and implicit time of the a- and b-waves (*x ± s*)

scotopic											
	0.01b (ms)	0.01b (***μv***)	3.0a (ms)	3.0a (***μv***)	3.0b (ms)	3.0b (***μv***)	OPS(***μv***)	10.0a (ms)	10..0a (***μv***)	10.0b (ms)	10..0b (***μv***)
Control	67.12 ±7.61	98.21 ±40.87	21.92 ±2.39	22.97 ±8.18	47.17 ±3.76	123.55 ±42.32	59.36 ±23.23	14.25 ±0.87	104.98 ±27.45	37.58 ±2.54	250.09 ±84.50
LD	69.6 ±14.23	57.31 ±30.04	26.6 ±5.36	17.85 ±6.31	52.2 ±4.57	98.5 ±21.53	43.54 ±24.87	16.10 ±4.36	99.72 ±34.46	42.70 ±3.02	167.42 ±46.87
LC	70 ±12.73	105.21 ±39.66∗	22.25 ±1.98	26.93 ±27.84∗	57.38 ±6.41	106 ±23.24	46.5 ±30.11	15.38 ±2.50	117.58 ±21.12	43.63 ±5.90	232.71 ±65.92
HC	70.33 ±17.85	126.43 ±61.09 **	22.5 ±4.93	39.017 ±14.39 ∗ ∗	37.57 ±20.13	138.57 ±42.17	60.68 ±21.31	14.33 ±1.37	116.28 ±24.18	41 ±3.58	241.17 ±52.20
photopic											
	3.0a(ms)	3.0a(***μv***)	3.0b(ms)	3.0b(***μv***)							
Control	20.08 ±2.78	4.94 ±2.90	43.58 ±3.75	46.99 ±12.52							
LD	24.9 ±3.84	3.113 ±1.88	46.8 ±5.55	46.54 ±24.37							
LC	22.75 ±2.87	3.93 ±1.27	45.13 ±2.29	52.48 ±11.84							
HC	20.67 ±3.82	5.48 ±1.37 *	40.83 ±2.40	53.85 ±15.02	`						

Amplitude and implicit time of a, b, and Ops of each group under the scotopic and photopic conditions. The data were analysed using ANOVA; ^*^P < 0.05, ^**^P < 0.01

#### FFA

**Fig. 8:**
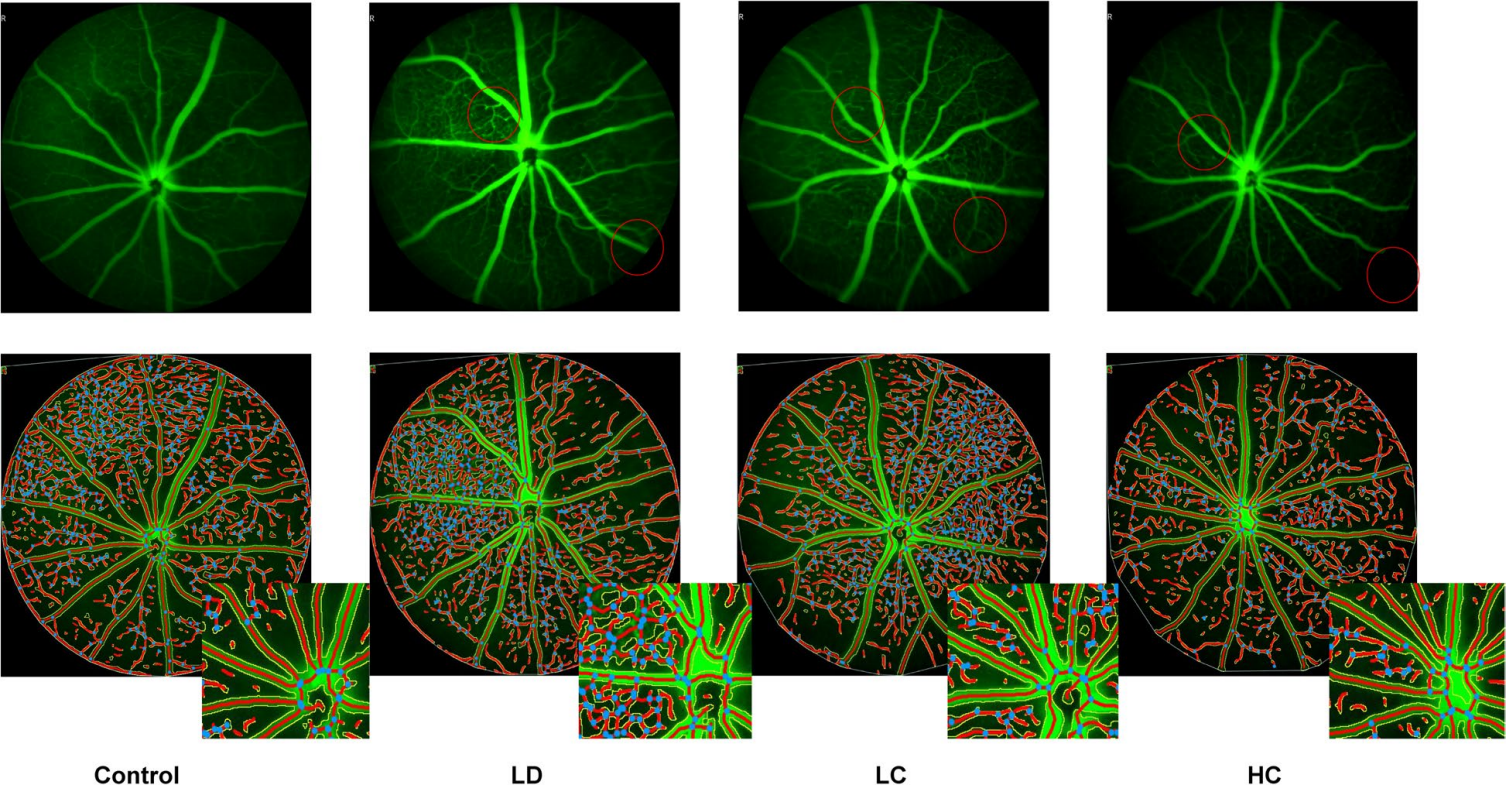
FFA images of the central fundus at the arteriovenous phase. With the optic disk at the centre position, FFA conducted at the arteriovenous phase showed that the intensity of the microvessels between 2 and 4 mm in diameter obviously increased in the LD group. The amplified detail red circle shows the vessel area percentage and the total number of junctions

**Fig. 9:**
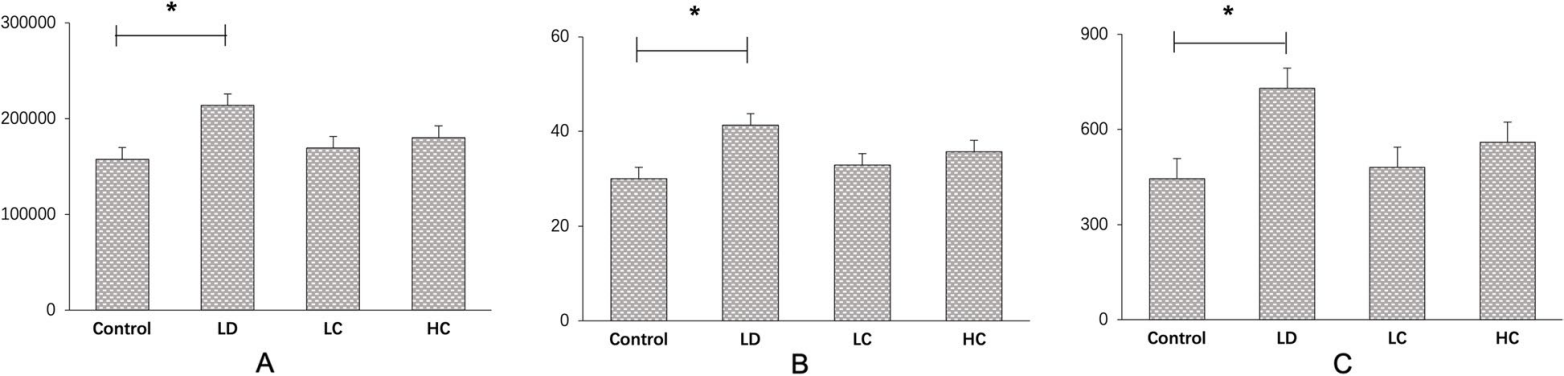
Comparison of the microvessels analysed using the AngioTool software. After processing the data using the AngioTool software and the parameters collected and analysed using the SPSS software, there were no significant differences found in the ANOVA results between the four groups; however, independent samples *t*-test showed that the LD group had a remarkable increase compared with the control group (^*^P < 0.05). **a**: vessel area, **b**: vessel area percentage, **c**: total number of junctions

The vessel area between 2 and 4 mm increased by 35% in the LD group compared with that in the control group, while that in the LC and HC groups decreased by 20.8 and 15.7%, respectively. The total number of junctions increased by 64.4% in the LD group compared with that in the control group; the total number in the LC and HC groups decreased by 34.2 and 23.3%, respectively, compared with that in the LD group. These parameters showed significant changes between the LD and control groups in the independent samples *t*-test (*P* < 0.05), as presented in Figs. 8 and 9.

#### Apoptosis rate of the retina as measured in the TUNEL assay

**Fig. 10:**
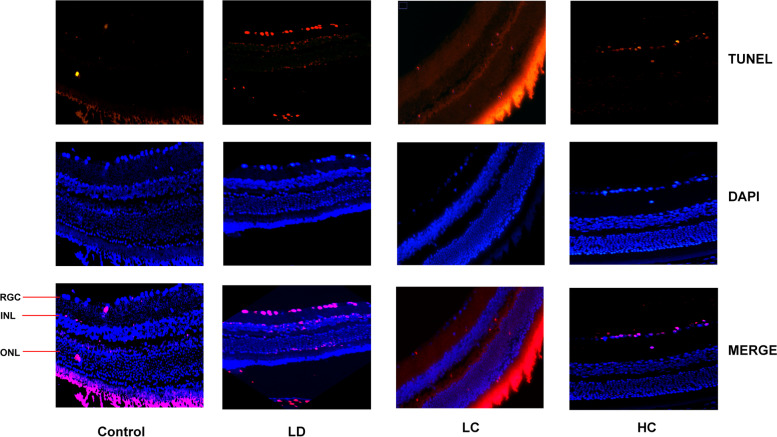
Apoptosis in the TUNEL assay. The number of apoptotic cells increased distinctly in each cell layer of the retina in the LD group. The apoptosis rate decreased obviously in the LC and HC groups, especially in the RGC layer

**Fig. 11:**
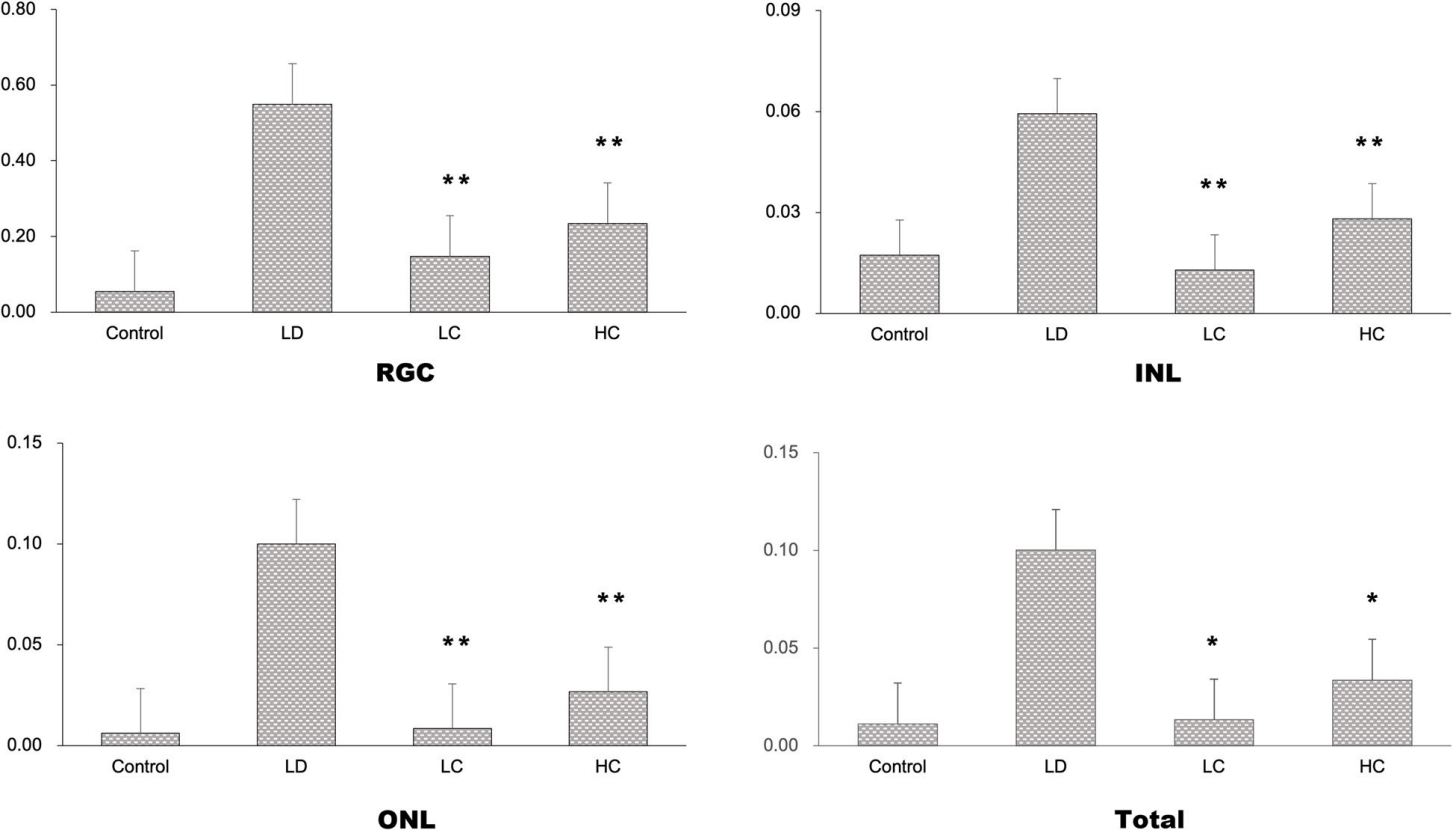
Comparison of the apoptosis of the cell layers. Using the ImageJ software, we counted the RGC, INL, ONL, and total number of apoptotic and normal cells. The SPSS software was used to analyse the apoptosis rate for the RGC (**a**), INL (**b**), ONL (**c**), and total cells (**d**) in each group. The apoptosis rate for the RGC in the LC and HC groups was much lower than that in the LD group (^**^P < 0.01); similar findings were observed for the INL (^**^P < 0.01). There was no significant change in the ONL apoptosis rate. The total apoptosis rate decreased significantly in the LC and HC groups (^**^P < 0.01)

The apoptosis rate markedly increased in the LD group, but decreased in the LC and HC groups (Fig. 10). The apoptosis rates of the RGCs, INL, and ONL were calculated separately (Fig. 11). The analysis showed significant differences in the apoptosis rate of the RGCs, INL, and entire retina between the four groups. The apoptosis rate of the RGCs, INL, and ONL in the LD group was much higher than that in the control group, while that in the LC and HC groups significantly improved. The RGC apoptosis rate in the LC group was 73.19%, while that in the HC group was 57.29% lower than that in the LD group (*P* < 0.01). The INL apoptosis rate in the LC group was 78.33%, while that in the HC group was 57.23% lower than that in the LD group (*P* < 0.01). The ONL apoptosis rate in the LC group was 91.39%, while that in the HC group was 73.18% lower than that in the LD group (*P* < 0.01).

#### Expression of NF-кB and TNF-α

**Fig. 12:**
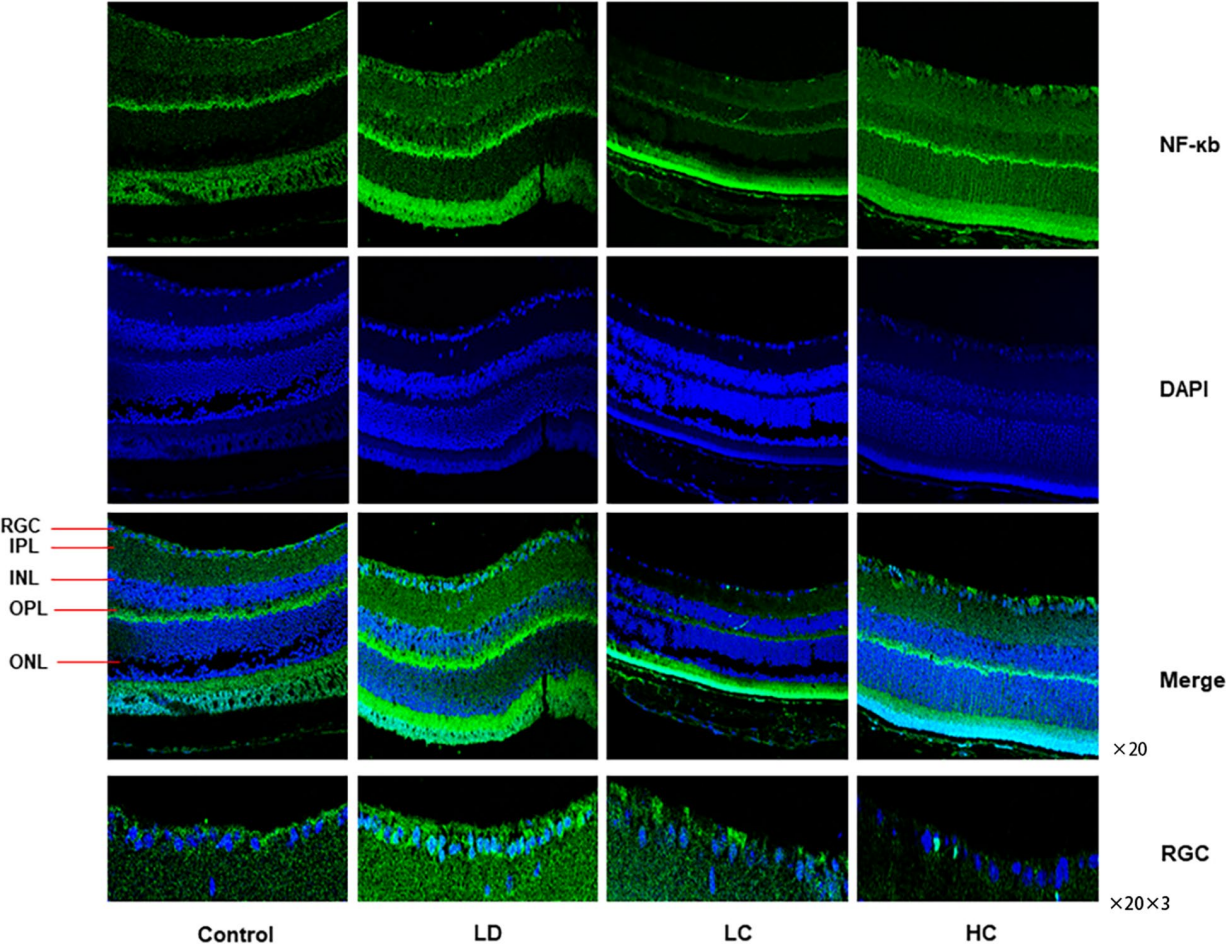
Immunofluorescence staining of NF-κB. The expression of NF-κB in the nucleus was enhanced in the retina in the LD group. NF-κB was mainly expressed in the nucleus of the retina, which was amplified three times by the RGC layer. The expression decreased in the LC and HC groups

**Fig. 13:**
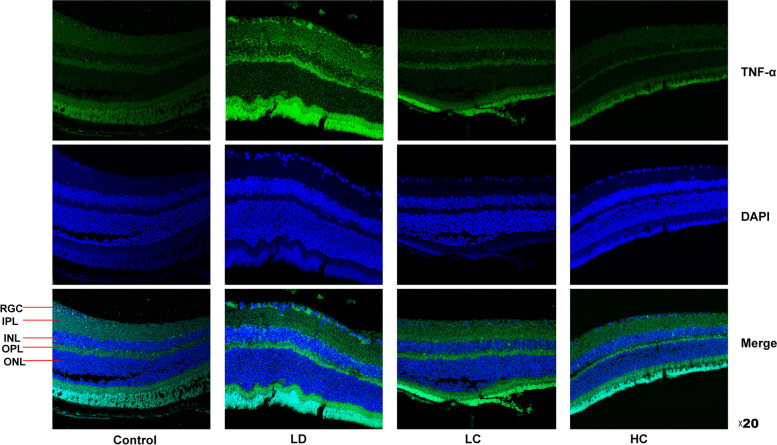
Immunofluorescence staining of TNF-α. The expression of TNF-α in the cytoplasm of the retinal cells significantly increased in the LD group but decreased in the LC and HC groups

**Fig. 14:**
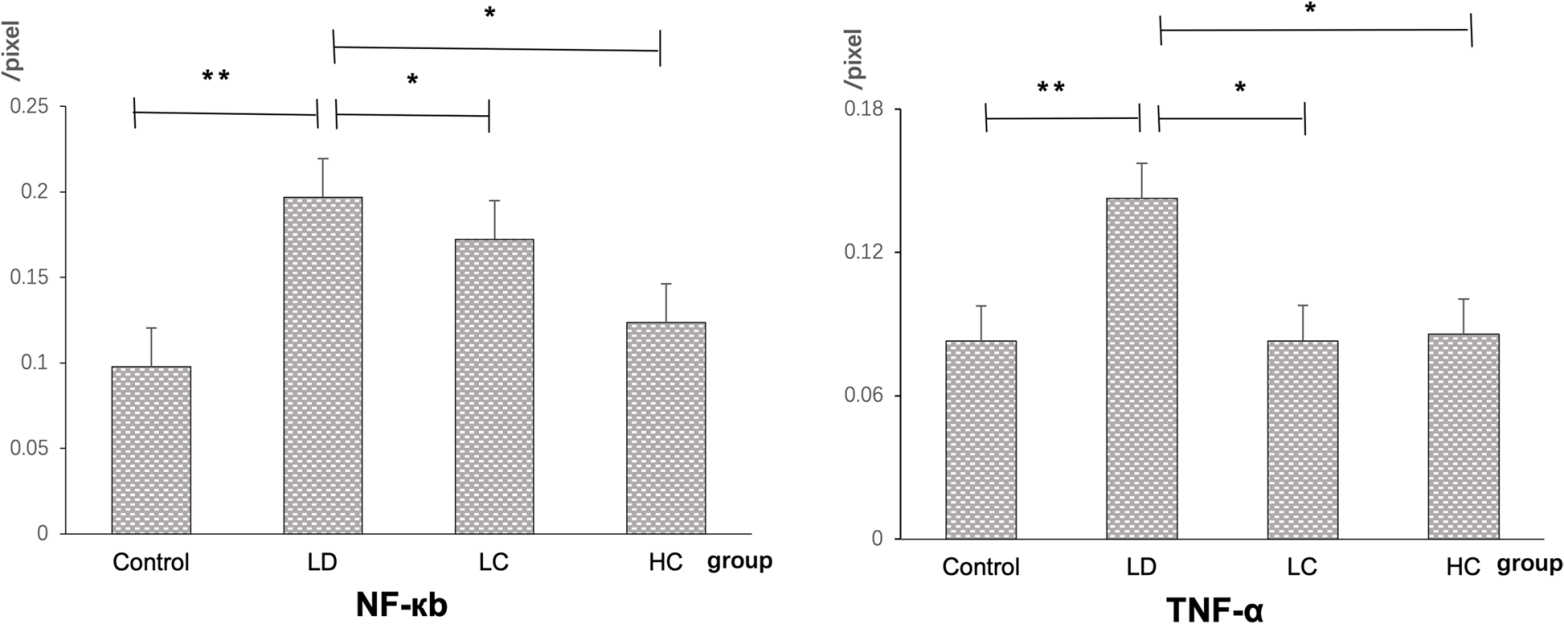
Comparison of the expression of NF-κB and TNF-α fluorescence density values between the groups. The expression of NF-кB in the nucleus was 111.1% higher in the LD group than in the control group but was 10.52% lower in the LC group and 36.84% lower in the HC group. The expression of TNF-α was 72.29% higher in the LD group than in the control group but was 41.96% in the LC group and 40.56% in the HC group (*P* < 0.05)

The expression rate of NF-кB localised to the nucleus was 111.1% higher in the LD group than in the control group, but was 10.52% lower in the LC group and 36.84% lower in the HC group than in the LD group. The expression rate of TNF-α was 72.29% higher in the LD group than in the control group, but was 41.96 and 40.56% in the LC and HC groups, respectively, compared with that in the LD group (Figs. 12, 13, 14) (*P* < 0.05).

#### Enzymatic activities of SOD, CAT, and GSH-Px

**Fig. 15:**
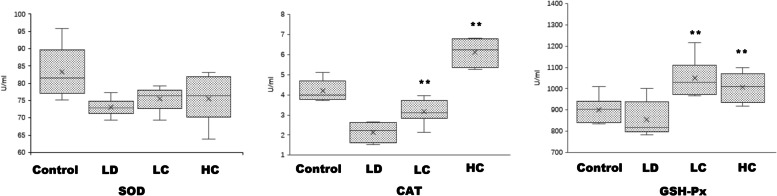
Enzymatic activities of SOD, CAT, and GSH-Px. The SOD (**a**), CAT (**b**), and GSH-Px (**c**) activities showed a large increase in the LC and HC groups, especially the CAT and GSH-Px activities

After continuous intragastric administration of the chrysanthemum extract, the enzymatic activity of SOD increased, with a mean value of 75.50 ± 3.2 U/mL in the LC group and 75.57 ± 6.33 U/mL in the HC group. The enzymatic activities of CAT and GSH-Px showed a more obvious increasing tendency, with a mean value of 1048.61 ± 85.18 U/mL in the LC group (*P* < 0.01) and 1005.56 ± 65.73 U/mL (*P* < 0.01) in the HC group (Fig. 15 and Table 3).

**Table 3:** Enzymatic activities of SOD, CAT, and GSH-Px

	SOD (U/ml)	CAT (U/ml)	GSH-Px (U/ml)
Control	82.36 ±6.90	4.19 ±0.49	901.39 ±58.02
LD	73.01 ±2.39	2.15 ±0.45	855.56 ±77.53
LC	75.50 ±3.20	3.16 ±0.57 ∗ ∗	1048.61 ±85.18 ∗ ∗
HC	75.57 ±6.33	6.12 ±0.62 ∗ ∗	1005.56 ±65.73 ∗ ∗

All the data are expressed as^−^*x ± s.* ^*^P < 0.05, ^**^P < 0.01

## Discussion

### Establishment of the model

Light damage induction, which has been used to establish an oxidative stress model in mice for more than 40 years, leads to photoreceptor destruction and inflammatory factor accumulation [20]. The acute or chronic approach to light damage is commonly used by researchers, and previous studies have shown that the development of AMD is related to subacute inflammation [21–23]. Therefore, strong light irradiation for a moderate period is feasible for modelling. In our experiments, a damaged structure of the RPE and photoreceptors was observed, which manifested as an arch formed with highly reflected signals on OCT that were very similar to what has been observed in AMD; similar outcomes were also shown on HE staining.

### Relationship between ROS production and retinal damage

Excessively powerful or long-term exposure to light leads to retinal phototoxic reactions, which produce a quantity of ROS that are considered the main metabolites of oxidative stress and are known to cause oxidative damage reactions [24, 25]. A recent clinical study found that IS/OS is less detailed for dividing the retinal layer than previously thought. New, more detailed zones are known as the MZ and EZ; these zones, particularly the EZ, have been shown to correlate with the inner segment ellipsoid, which contains a large concentration of mitochondria [26]. When excess ROS cannot be resolved by the mitochondria, some non-degradable lysosomal substances accumulate, which results in the formation of lipofuscin. These substances include dysfunctional mitochondria and outer segments of engulfed apoptotic photoreceptor cells [25]. The deposition of lipofuscin in the early stages does not cause abnormal cell metabolism. However, RPE cells are permanent cells in the body, and the accumulated lipofuscin cannot be diluted by cell proliferation. Long-term accumulation of intracellular lipofuscin affects the function of the mitochondria and lysosomes, as well as the proteolysis system [27, 28]. This decreased proteolytic capacity leads to the accumulation of extracellular oxidised proteins, which results in drusen formation [29] and may cause apoptosis to increase in the retina. Some studies have found that abnormal autophagy and DNA damage response (DDR) are important in the mechanism of AMD, and the main reason for this is related to an increase in ROS levels [30]. Therefore, oxidative stress is not only an important factor in cell senescence but also leads to the formation of a malignant pathological circulation mechanism of AMD. In our study, we observed increased ROS production and decreased viability induced by light damage in the RPE cells. In addition, damage to the EZ, OSP, and RPE layers and increased apoptosis in the retina were observed on OCT and HE staining. Based on these results, we conclude that the antioxidant process is important for preventing AMD development in the retina.

### Effect of the chrysanthemum extract on the oxidatively stressed retina

Among the remarkable achievements of traditional Chinese medicine in the study of antioxidants, chrysanthemum is a plant of the Asteraceae family that is characterised as sweet, bitter, and slightly cold and is used to treat the lung and liver meridians. It has the ability to dissipate heat and protect the eye from decreasing vision and fatigue. The chrysanthemum extract is rich in flavonoids and polysaccharides [31]. Wu et al. demonstrated that wild chrysanthemum eye drops had a protective effect against dry eye in humans [32], improving light and moderate levels of dry eye in patients. Hollyfield et al. proved that oxidative stress plays a key role in AMD, inflammatory response, and retinal morphological abnormalities caused by oxidative stress markers, such as CEP and MDA, which are very similar to those observed in AMD [33].

In this study, the concentration of flavonoids that the source materials contributed to the antioxidants was no less than 5%. The electrophysiological function indicated on ERG and the morphology of the retina displayed on OCT and HE staining significantly improved after treatment with the chrysanthemum extract. These results are similar to those reported by Kim et al., which are consistent with the conclusion that chrysanthemum can improve the loss of neuronal viability induced by MPP+, reduce the apoptosis rate, and increase the expression of Bcl-2 [13]. Sun et al. also found that wild chrysanthemum extracts can reduce the level of ROS in cells and protect against ultraviolet damage and skin photo ageing in vitro [34]. In contrast, the microvessel area, vessel area percentage, and total number of junctions as measured on FFA and analysed using the AngioTool software indicated that there was an increase in microvessel production in the LD group, which may be related to neovascularisation. Although there was a decreasing tendency in the vessel area, vessel area percentage, and total number of junctions in the LC and HC groups, these changes were not significant.

### Decreased NF-кB and TNF-α expression in the LC and HC groups

**Fig. 16:**
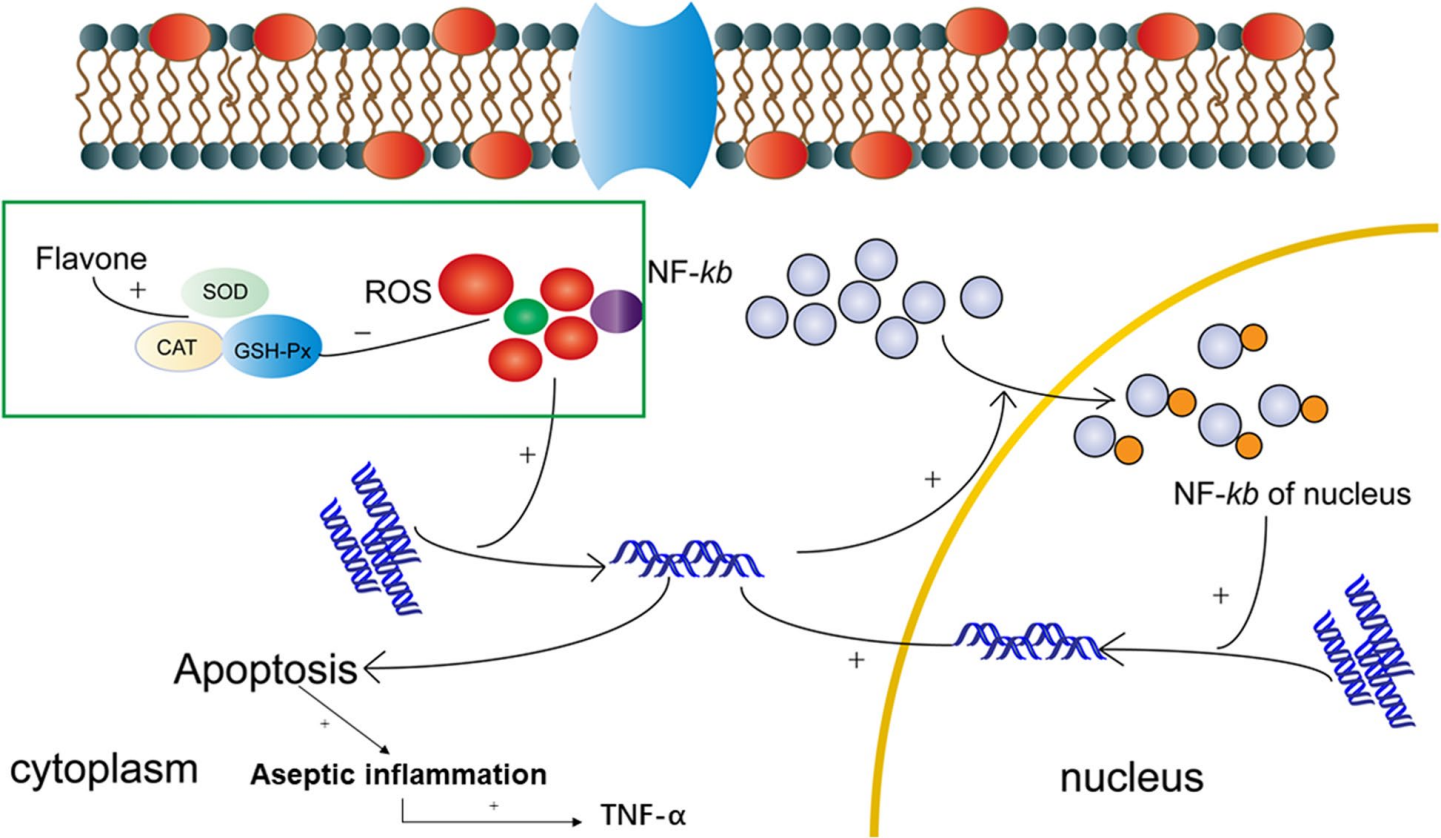
Mechanism of the protective effect of the chrysanthemum extract. ROS production could activate NF-кB in the nucleus and increase apoptosis of the retina through the DDR pathway. ROS production could simultaneously induce apoptosis through DDR via the mitochondrial pathways. There may be aseptic inflammation when too many apoptotic cells occur, which accelerates TNF-α secretion in the retina. The flavonoid molecular materials from the chrysanthemum extract could increase SOD, CAT, and GSH-Px production, which could have an inhibitory effect on ROS production

Numerous studies have shown that chrysanthemums have anti-inflammatory effects [10, 35]. ROS production can activate NF-кB in the nucleus and increase apoptosis in the retina through the DDR pathway. There is also aseptic inflammation that occurs with the activation of macrophages when too many apoptotic cells are produced and TNF-α is raised in tissues [36]. In our experiment, the expression rates of NF-кB localised in the nucleus and TNF-α in the LD group were much higher than those in the control group and decreased in the LC and HC groups. The chrysanthemum extract reduced ROS production and the apoptosis rate in the retinal tissue; the reduced apoptosis rate attenuated aseptic inflammation, which induced a decrease in TNF-α expression in our experiment (Fig. 16).

### Increased SOD, CAT, and GSH-Px activities in the blood after chrysanthemum extract administration

The activities of the antioxidant enzymes SOD, CAT, and GSH-Px increased after treatment with the chrysanthemum extract. The changes in the CAT and GSH-Px activities were obvious. However, the SOD activity increased, but with no significance, which may have been related to the number of samples included. Therefore, we speculated that chrysanthemum extracts are effective in treating retinal damage induced by light and may play a role in increasing antioxidant enzymatic activities and reducing ROS levels (Fig. 15).

## Conclusions

In our study, increased ROS production was observed in the light-damaged RPE cells, which damaged the structure and function and increased apoptosis in the light-damaged retinas. Preventive administration of the chrysanthemum extract improved both the function and morphology of the retina, reduced the production of ROS by increasing the SOD, CAT, and GSH-Px activities, decreased the expression of NF-кB and apoptosis, and reduced the expression TNF-α and aseptic inflammation; this finding indicates that chrysanthemum has a potential as a traditional Chinese medicine for protecting the retina from light-induced damage, which is related to the pathogenesis of AMD.

## Data Availability

The datasets analysed during the current study are available from the corresponding authors on reasonable request.

## Abbreviations

AMD: Age-related macular degeneration
ROS: Reactive oxygen species
SOD: Superoxide dismutase
CAT: Catalase
GSH-Px: Glutathione peroxidase
ERG: Electroretinogram
OCT: Optical coherence tomography
FFA: Fluorescein fundus angiography
OSP: Outer segment of the photoreceptors
EZ: Ellipsoid zone
MZ: Myoid zone
INL: Inner nuclear layer
ONL: Outer nuclear layer
RPE: Retinal pigment epithelium
LD group: Light-damaged group
LC group: Low-dose chrysanthemum extract group
HC group: High-dose chrysanthemum extract group
HE: Haematoxylin and eosin
DDR: DNA damage response

## References

[1] Wong WL, Su X, Li X, Cheung CM, Klein R, Cheng CY (2014). Global prevalence of age-related macular degeneration and disease burden projection for 2020 and 2040: a systematic review and meta-analysis. Lancet Glob Health.

[2] Lim LS, Mitchell P, Seddon JM, Holz FG, Wong TY (2012). Age-related macular degeneration. Lancet..

[3] Jager RD, Mieler WF, Miller JW (2008). Age-related macular degeneration. N Engl J Med.

[4] Cano M, Thimmalappula R, Fujihara M, Nagai N, Sporn M, Wang AL (2010). Cigarette smoking, oxidative stress, the anti-oxidant response through Nrf2 signaling, and age-related macular degeneration. Vis Res.

[5] Chakravarthy U, Wong TY, Fletcher A, Piault E, Evans C, Zlateva G (2010). Clinical risk factors for age-related macular degeneration: a systematic review and meta-analysis. BMC Ophthalmol.

[6] Jonasson F, Fisher DE, Eiriksdottir G, Sigurdsson S, Klein R, Launer LJ (2014). Five-year incidence, progression, and risk factors for age-related macular degeneration: the age, gene/environment susceptibility study. Ophthalmology..

[7] Li R, Liu Y, Xie J, Huang X, Zhang L, Liu H (2019). Sirt3 mediates the protective effect of hydrogen in inhibiting ROS-induced retinal senescence. Free Radic Biol Med.

[8] Zheng L, Van Labeke MC (2017). Chrysanthemum morphology, photosynthetic efficiency and antioxidant capacity are differentially modified by light quality. J Plant Physiol.

[9] Jing CL, Huang RH, Su Y, Li YQ, Zhang CS. Variation in chemical composition and biological activities of flos chrysanthemi indici essential oil under different extraction methods. Biomolecules. 2019;9(10). 10.3390/biom9100518 PMC6843213 31546663

[10] Zhang N, He Z, He S, Jing P (2019). Insights into the importance of dietary chrysanthemum flower (Chrysanthemum morifolium cv. Hangju)-wolfberry (Lycium barbarum fruit) combination in antioxidant and anti-inflammatory properties. Food Res Int.

[11] Guo H, Yuan Q, Fu Y, Liu W, Su YH, Liu H, et al. Extraction optimization and effects of extraction methods on the chemical structures and antioxidant activities of polysaccharides from snow chrysanthemum (*Coreopsis tinctoria*). Polymers. 2019;11(2). 10.3390/polym11020215 PMC6419038 30960199

[12] Tanaka J, Nakanishi T, Ogawa K, Tsuruma K, Shimazawa M, Shimoda H (2011). Purple rice extract and anthocyanidins of the constituents protect against light-induced retinal damage in vitro and in vivo. J Agric Food Chem.

[13] Kim IS, Koppula S, Park PJ, Kim EH, Kim CG, Choi WS (2009). Chrysanthemum morifolium Ramat (CM) extract protects human neuroblastoma SH-SY5Y cells against MPP+-induced cytotoxicity. J Ethnopharmacol.

[14] Cao X, Guo Y, Wang Y, Wang H, Liu D, Gong Y (2020). Effects of high-fat diet and Apoe deficiency on retinal structure and function in mice. Sci Rep.

[15] Chung YR, Choi JA, Koh JY, Yoon YH (2017). Ursodeoxycholic acid attenuates endoplasmic reticulum stress-related retinal pericyte loss in streptozotocin-induced diabetic mice. J Diabetes Res.

[16] Zudaire E, Gambardella L, Kurcz C, Vermeren S (2011). A computational tool for quantitative analysis of vascular networks. PLoS One.

[17] Liu Y, Li R, Xie J, Hu J, Huang X, Ren F (2018). Protective effect of hydrogen on sodium iodate-induced age-related macular degeneration in mice. Front Aging Neurosci.

[18] Kyrylkova K, Kyryachenko S, Leid M, Kioussi C (2012). Detection of apoptosis by TUNEL assay. Methods Mol Biol.

[19] Tachtsis B, Whitfield J, Hawley JA, Hoffman NJ (2020). Omega-3 polyunsaturated fatty acids mitigate palmitate-induced impairments in skeletal muscle cell viability and differentiation. Front Physiol.

[20] Yamashita H, Horie K, Yamamoto T, Nagano T, Hirano T (1992). Light-induced retinal damage in mice. Hydrogen peroxide production and superoxide dismutase activity in retina. Retina..

[21] Ahmed CM, Biswal MR, Li H, Han P, Ildefonso CJ, Lewin AS (2016). Repurposing an orally available drug for the treatment of geographic atrophy. Mol Vis.

[22] Song D, Song Y, Hadziahmetovic M, Zhong Y, Dunaief JL (2012). Systemic administration of the iron chelator deferiprone protects against light-induced photoreceptor degeneration in the mouse retina. Free Radic Biol Med.

[23] Song D, Song J, Wang C, Li Y, Dunaief JL (2016). Berberine protects against light-induced photoreceptor degeneration in the mouse retina. Exp Eye Res.

[24] Hanus J, Anderson C, Wang S (2015). RPE necroptosis in response to oxidative stress and in AMD. Ageing Res Rev.

[25] Mitter SK, Song C, Qi X, Mao H, Rao H, Akin D (2014). Dysregulated autophagy in the RPE is associated with increased susceptibility to oxidative stress and AMD. Autophagy..

[26] Cuenca N, Ortuño-Lizarán I, Sánchez-Sáez X, Kutsyr O, Albertos-Arranz H, Fernández-Sánchez L (2020). Interpretation of OCT and OCTA images from a histological approach: clinical and experimental implications. Prog Retin Eye Res.

[27] Boyer NP, Higbee D, Currin MB, Blakeley LR, Chen C, Ablonczy Z (2012). Lipofuscin and N-retinylidene-N-retinylethanolamine (A2E) accumulate in retinal pigment epithelium in absence of light exposure: their origin is 11-cis-retinal. J Biol Chem.

[28] Sitte N, Huber M, Grune T, Ladhoff A, Doecke WD, Von Zglinicki T (2000). Proteasome inhibition by lipofuscin/ceroid during postmitotic aging of fibroblasts. FASEB J.

[29] Ferrington DA, Sinha D, Kaarniranta K (2016). Defects in retinal pigment epithelial cell proteolysis and the pathology associated with age-related macular degeneration. Prog Retin Eye Res.

[30] Hyttinen JMT, Błasiak J, Niittykoski M, Kinnunen K, Kauppinen A, Salminen A (2017). DNA damage response and autophagy in the degeneration of retinal pigment epithelial cells-implications for age-related macular degeneration (AMD). Ageing Res Rev.

[31] Long T, Xu Y, Kong W, Xiao WP, Xu LY. Simultaneous determination and comparison of phenolic bioactives among three main kinds of edible chrysanthemums. J Chromatogr Sci. 2022. 10.1093/chromsci/bmac009 35169829

[32] Wu JL, Liu ZG, Jin M, Liu J, Li Y, Bi HS (2021). A multicenter, randomized, double-masked, placebo-controlled trial of compound wild chrysanthemum eye masks for mild and moderate dry eye. Zhonghua Yan Ke Za Zhi.

[33] Renganathan K, Gu J, Rayborn ME, Crabb JS, Salomon RG, Collier RJ (2013). CEP biomarkers as potential tools for monitoring therapeutics. PLoS One.

[34] Sun S, Jiang P, Su W, Xiang Y, Li J, Zeng L (2016). Wild chrysanthemum extract prevents UVB radiation-induced acute cell death and photoaging. Cytotechnology..

[35] Chen JM, Wang T, Guo QS, Li HW, Zuo L, Zou QJ (2021). Comprehensive antioxidant and anti-inflammatory activity of alcohol extracts from Chrysanthemum indicum in different areas based on entropy weight and TOPSIS methodology. Zhongguo Zhong Yao Za Zhi.

[36] Devarajan G, Niven J, Forrester JV, Crane IJ (2016). Retinal pigment epithelial cell apoptosis is influenced by a combination of macrophages and soluble mediators present in age-related macular degeneration. Curr Eye Res.

